# Cesium Lead Halide Perovskite Nanocrystals Assembled in Metal‐Organic Frameworks for Stable Blue Light Emitting Diodes

**DOI:** 10.1002/advs.202105850

**Published:** 2022-03-15

**Authors:** Hsinhan Tsai, Hsin‐Hsiang Huang, John Watt, Cheng‐Hung Hou, Joseph Strzalka, Jing‐Jong Shyue, Leeyih Wang, Wanyi Nie

**Affiliations:** ^1^ Center for Integrated Nanotechnologies Los Alamos National Laboratory Los Alamos NM 87545 USA; ^2^ Department of Chemistry University of California Berkeley Berkeley CA 94720 USA; ^3^ Center for Condensed Matter Sciences National Taiwan University Taipei 10617 Taiwan; ^4^ Department of Material Science and Engineering National Taiwan University Taipei 10617 Taiwan; ^5^ Research Center for Applied Science Academia Sinica Taipei 11529 Taiwan; ^6^ X‐Ray Science Division Argonne National Laboratory Argonne IL 60439 USA; ^7^ Center of Atomic Initiative for New Materials National Taiwan University Taipei 10617 Taiwan

**Keywords:** blue light emitting diodes (LEDs), inorganic perovskite nanocrystals, metal‐organic frameworks

## Abstract

All inorganic cesium lead trihalide nanocrystals are promising light emitters for bright light emitting diodes (LEDs). Here, CsPb(BrCl)_1.5_ nanocrystals in metal‐organic frameworks (MOF) thin films are demonstrated to achieve bright and stable blue LEDs. The lead metal nodes in the MOF thin film react with Cs‐halide salts, resulting in 10–20 nm nanocrystals. This is revealed by X‐ray scattering and transmission electron microscopy. Employing the CsPbX_3_‐MOF thin films as emission layers, bright deep blue and sky‐blue LEDs are demonstrated that emit at 452 and 476 nm respectively. The maximum external quantum efficiencies of these devices are 0.72% for deep blue LEDs and 5.6% for sky blue LEDs. More importantly, the device can maintain 50% of its original electroluminescence (*T*_50_) for 2.23 h when driving at 4.2 V. Detailed optical spectroscopy and time‐of‐flight secondary ion mass spectroscopy suggest that the ion migration can be suppressed that maintains the emission brightness and spectra. The study provides a new route for fabricating stable blue light emitting diodes with all‐inorganic perovskite nanocrystals.

## Graphical abstract

A new strategy is demonstrated for integrating Cs‐based perovskites nanocrystal in metal‐organic framework (MOF) thin films with bright emissions from green to deep blue. The light emitting diodes made with MOF protect perovskite nanocrystal exhibit high performance electroluminescence with external quantum efficiencies of 5.6% and extended operation lifetime over 2 h.

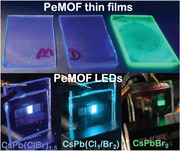

## 1 Introduction

Blue light emitting diodes (LEDs) are important technologies for display panel, solid state lighting, photolithography etc.^[^ ^1^ ^]^ However, only few semiconductors can be used for high efficiency blue LEDs.^[^ ^2^ ^]^ Perovskite materials^[^ ^3^, ^4^ ^]^ and nanocrystals^[^ ^4^, ^5^, ^6^, ^7^, ^8^, ^9^ ^]^ are recent popular candidates for low‐cost, high performance LEDs^[^ ^5^, ^10^, ^11^ ^]^ and high‐energy detector.^[^ ^7^, ^12^, ^13^ ^]^ Among the rich perovskite material family, cesium lead halide perovskites have received extensive attention because of their outstanding emission quantum yield^[^ ^4^, ^8^, ^14^ ^]^ and high heat tolerance.^[^ ^12^, ^15^ ^]^ The emission color can be easily tuned by substituting the halides in CsPbBr_3_ with chloride where blue emissions have been achieved. Besides, hot injection method has been a commonly used synthetic approach for fabricating high quality all inorganic perovskite nanocrystals.^[^ ^4^, ^16^ ^]^ It is also discovered that proper choices of ligands are critical for surface trap passivation to achieve near unity emission yield.^[^ ^17^ ^]^ These pioneer demonstrations have greatly enriched the material choices for the blue LEDs. Utilizing the all inorganic perovskite nanocrystals, Song et al achieved an early breakthrough for blue perovskite LEDs, that showed a promising external quantum efficiency (EQE) of 0.07% with a turn on voltage of 5.1 V.^[^ ^9^ ^]^ Further, it is found that by incorporating organic cations like phenylethylamine, the emission quantum yield of the CsPbX_3_ blue emitter can be further improved, where >10% EQE have been demonstrated for a sky‐blue LED.^[^ ^18^, ^19^ ^]^ More recently, Hou et al have demonstrated Mn doping in the perovskite nanocrystals and use that for a high efficiency deep blue LED with an EQE over 2%.^[^ ^20^ ^]^

While constant efforts have been devoted in boosting the performance of blue perovskite LEDs,^[^ ^11^, ^18^, ^19^, ^20^, ^21^, ^22^ ^]^ the operational stability of the blue LEDs are not fully addressed. It is believed that trap could form during LEDs operation that can cause a decay in the electroluminescence.^[^ ^23^, ^24^ ^]^ In mixed halide perovskite nanocrystals, the material instability under operational conditions, i.e., ion migration under constant electrical bias, further worsens the degradation problem. It is shown that under constant laser irradiation and current injection conditions, mixed halide CsPb(Br*_x_* Cl_3−_ *_x_*) nanocrystals undergo phase segregation that shift the emission spectra,^[^ ^25^ ^]^ which is attributed to the field induced ion migration that causes phase segregation.^[^ ^26^ ^]^ In particular, the Cl and Br ions diffuse at a relatively faster rate than the I ions, which make it more challenging to stabilize the blue perovskite LEDs.^[^ ^27^, ^28^ ^]^ Very recently, additives like ZnBr^[^ ^29^ ^]^ or organic agents^[^ ^22^, ^30^ ^]^ have been incorporated in the perovskite emission layer that extended the blue LEDs’ lifetime.

Here we introduce a new nanocrystalline CsPbX_3_ thin film fabrication method for stable blue LEDs. By introducing CsX (X = Br or Cl) into the metal‐organic framework (MOF)^[^ ^5^, ^31^, ^32^ ^]^ thin film containing Pb metal nodes (Pb‐MOF), 10–20 nm CsPbX_3_ nanocrystals can form that are surrounded by the MOF matrix. Revealed by transmission electron microscopy, instead of alloying the Br and Cl ions in a single particle, individual CsPbX_3_ nanocrystals are observed inside the MOF matrix. By tuning the relative percentage of the Cl and Br in the precursor, we obtain both sky blue and deep blue emissions in perovskites‐MOF (PeMOF). LEDs are fabricated using deep blue and sky‐blue emitters that deliver external quantum efficiencies of 5.6% and 0.7% respectively. The LEDs can maintain 50% of the original brightness (*T*_50_) after 8000 s and 300 s under low and high driving voltages respectively. After a detailed power dependent photoluminescence stability investigation, we find the Cs‐PeMOF's emission can sustain much higher laser power irradiation against light induced ion migration comparing to that of the CsPbBr_3−_ *_x_* Cl*_x_* thin film. The time‐of‐flight secondary‐ion mass spectroscopy (ToF‐SIMS) depth profile shows uniform components distribution inside the Cs‐PeMOF film after intense voltage and injection current stressing, verifying the limited ion‐migration dynamic within the PeMOF nanocomposite that enables the superior operational stability. The results demonstrated herein shed light on stabilizing the perovskite nanocrystals in PeMOF structure for blue LEDs.

## 2 Results and Discussion

**Figure 1:**
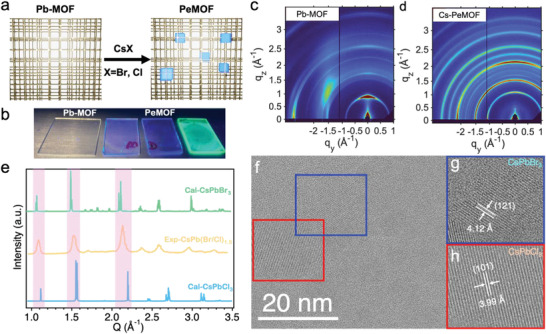
a) Schematic illustration of the PeMOF fabrication process and b) the obtained thin films under UV lamp illumination. GIWAXS maps comparison for c) Pb‐MOF and d) Cs‐PeMOF. e) Line‐cut profile extracted from (d) and compared with the simulated XRD patterns for CsPbBr_3_ and CsPbCl_3_ structures. f) Transmission electron micrograph of the Cs‐PeMOF thin film and g,h) zoomed‐in view of a nanocrystal.

MOF materials have unique characteristic properties such as permanently porous structure, structure diversity and tunable functionality with thermal and chemical stability.^[^ ^32^ ^]^ In this study, we choose Pb‐MOF as the Pb source for perovskite nanocrystal synthesis in order to obtain nanocrystals embedded in the MOF matrix.^[^ ^33^ ^]^ **Figure** **1** illustrates the thin film fabrication process and the structure characterizations for Cs‐PeMOF thin films. As illustrated in Figure 1a, the Pb‐MOF thin film is first spin coated on the substrate and is baked at 100 °C for 30 min. A precursor containing CsX salts (i.e., CsCl or CsBr) is then spin coated on the Pb‐MOF thin film to convert the Pb metal core into CsPbX_3_ nanocrystals. As shown by the photograph in Figure 1b, the pristine MOF thin film does not have noticeable luminescence under UV lamp (254 nm) excitation, whereas the PeMOF thin films exhibit strong emission and the color is tunable by changing the halide composition (Cl/Br ratio = 1:1 (left), 1:2 (middle) and 0:3 (left)). The strong emission indicats the in‐situ nanocrystal formation after Pb‐MOF react with cations. In addition, we use synchrotron grazing incidence wide‐angle X‐ray scattering (GIWAXS) technique and high‐resolution transmission electron microscopy (HRTEM) to understand the crystalline structure and morphology of the obtained thin films.

The GIWAXS characterization profiles for Pb‐MOF and PeMOF thin films are shown in Figure 1c,d. In the Pb‐MOF thin film (Figure 1c), we observed a dominant scattering feature at 0.9 Å^–1^, indicating the structure has a d‐spacing of 1.43 nm, predominantly in the our‐of‐plane direction (perpendicular to the substrate). Besides, there is a ring‐like feature arising at 1.75 Å^–1^ which represents the pi‐pi stacking of the aromatic components^[^ ^34^ ^]^ in the MOF structure. Comparing to MOF thin film, the GIWAXS map of PeMOF confirmed the formation of nanocrystals CsPb(BrCl)_1.5_ but also exhibit much stronger crystallinity with partially Pb‐MOF features reserved (Figure S1, Supporting Information). We first extract the GIWAXS line‐cut (gold) from Figure 1d and compare that with the simulated CsPbBr_3_ (orthorhombic, green) and CsPbCl_3_ (tetragonal, blue) diffraction patterns in Figure 1e. Here the simulated diffraction patterns are based on the stable phase at room temperature. The dominant peaks are (101)/(121)/(202) planes of CsPbBr_3_ orthorhombic phase (green) and (100)/(101)/(200) planes for CsPbCl_3_ tetragonal phase (blue). As we look closely for the line‐cut for CsPb(BrCl)_1.5_, the dominant peaks are in between of CsPbBr_3_ and CsPbCl_3_ which suggested the formation of mix‐halides perovskites and followed d‐spacing evolution from Br to Cl. Moreover, by analyzing the peaks’ full width at half maximum corresponding to the CsPbX_3_ structure, and fit them by Scherrer equation, we obtain a mean size of crystalline domains of 19.7 nm.

We further performed the high‐resolution transmission electron microscopy (TEM) for Cs‐PeMOF thin films. Figure 1f shows the large view of a typical TEM image for CsPb(BrCl)_1.5_ nanocrystal in MOF thin film, from which we identify nanocrystals in the MOF matrix with a size of 10–20 nm, that are randomly distributed in the sample. 10–20 nm nanocrystals are found to be presence in most of the region in the samples revealed by more TEM images shown in the Supporting Information (Figure S2, Supporting Information). In addition, we took HRTEM images for a few typical individual nanocrystals and analyze the crystal structures by their lattice spacings and electron diffraction patterns shown in Figure 1g,h. Within the particles we analyzed, the nanocrystals that have alloy of mix‐halides in a single particle were not found, instead, we found the distribution of pure Cl and pure Br structures in the nanocrystals. For instance, Figure 1g matches with the (121) plane of the CsPbBr_3_ Orthorhombic Pnma structure, whereas the Figure 1h matches with the (101) plane of the CsPbCl_3_ tetragonal *P*4*mm* phase. More analysis is done with other individual crystals in Supporting Information (Figures S3–S6, Supporting Information) where crystals matching with either CsPbBr_3_ or CsPbCl_3_ are observed. In the GIWAXS line‐cut profile of the Cs‐PeMOF thin film, on the other hand, we did not observe the signature for individual phase co‐existing in the sample like a peak splitting. The discrepancy could be because of the broadened GIWAXS peaks for the nanocrystals where peak splitting is not observed. However, we could not exclude the fact that the TEM images examine the surface part of the crystals, where the alloyed crystals may locate in the bulk film.

**Figure 2:**
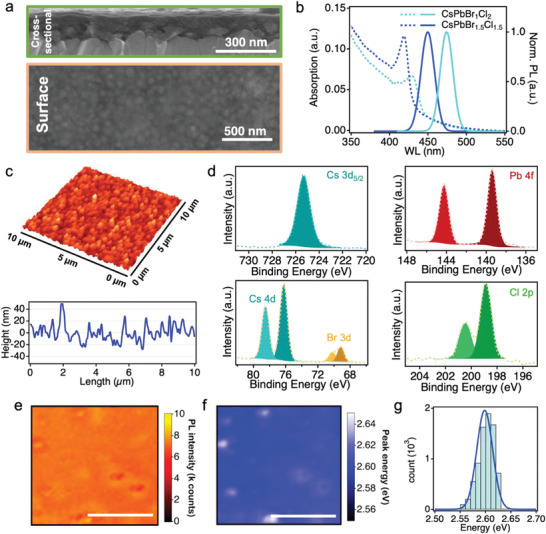
Thin film morphology and emission property characterizations. a) Surface and cross‐sectional SEM images of PeMOF thin film. b) Absorption and PL spectra for PeMOF with 33.3% Br and 50% Br in the precursor. c) AFM characterization (top) and corresponding line‐scan (bottom) of the as‐prepared Cs‐PeMOF. d) Characteristic XPS spectra of Cs, Pb, Br, and Cl elements acquired from the Cs‐PeMOF thin film. e) PL height map and f) peak energy map of a typical CsPb(BrCl)_1.5_ PeMOF thin film. Scale bars in e and f are 20 µm. g) The peak position distribution extracted from (f).

The film morphology, PL characteristic, and chemical composition of the Cs‐PeMOF films are carefully characterized and the results are shown in **Figure** **2**. The cross‐sectional SEM image (Figure 2a, top panel) verifies the successful fabrication of a uniform and compact 100–110 nm‐thick Cs‐PeMOF thin film. The surface morphology of the Cs‐PeMOF thin film is examined by the SEM top‐view image (Figure 2a, bottom panel). The surface of the Cs‐PeMOF thin film is largely amorphous, i.e., no crystalline grain features are observed. The roughness can be attributed to the aggregates of the Cs‐PeMOF nanocrystal clusters in the film, that are found to be uniformly distributed. The absorption and PL spectra of the Cs‐PeMOF thin films are plotted in Figure 2b, demonstrating a tunable PL emission enabled by the halide‐component engineering. When the Br/Cl content ratio is 1:1 in the Cs‐PeMOF thin film, its absorption edge is near 450 nm and the characteristic emission wavelength is centered around 475 nm. By elevating the Cl content to a Br/Cl ratio of 1:2, the absorption edge and the characteristic emission wavelength are blue shifted to around 430 and 450 nm, respectively, resulting in a deep‐blue PL emission of the Cs‐PeMOF thin film. A significant stokes shift can be observed in both spectra associated to the nanostructure formation.^[^ ^35^ ^]^ We further compare the PL line width (Figure S7, Supporting Information) and lifetime (Figure S8, Supporting Information) with pure Br and mix Br/Cl composition which suggest the material crystallinity and stability are comparable with different charger transfer process. The surface roughness of the thin film is examined by atomic force microscopy (AFM). Both the 3D image with corresponding line scan analysis are shown in Figure 2c indicate a reasonably uniform topography of the Cs‐PeMOF surface, featuring a height variation less than 50 nm. Besides, the surface roughness of 16 ± 3.4 nm can be obtained by surface line scan (Figure 2c bottom). The MOF topography is shown in Figure S9 in the Supporting Information, where 30 nm domains are observed. To verify the halide composition of CsPbX_3_ nanocrystals assembled in the MOF matrix, the elemental composition of a mix‐halide (Br and Cl) and pure bromide Cs‐PeMOF thin films are investigated using X‐ray photoelectron spectroscopy (XPS). According to Figure 2d, all the characteristic peaks, i.e., Cs 3d_5/2_, Cs 4d, Pb 4f, Br 3d, and Cl 2p, can be clearly identified in the XPS spectra obtained from the mix‐halide Cs‐PeMOF film, whilst no Cl 2p signal can be observed in the pure bromide Cs‐PeMOF thin film (Figure S10, Supporting Information). The pure and alloyed halide composition shown in the XPS spectra confirm that our solution‐based fabrication procedure enables an effective approach to modify the halide‐content of the Cs‐PeMOF nanocrystals, which is essential in achieving a tunable emission characteristic of the LEDs device.

To examine the emission uniformity in the film, we take the CsPb(BrCl)_1.5_ PeMOF thin film for spatial resolved photoluminescence measurement with a spatial resolution of 2 µm. The PL height and peak position maps are shown in Figure 2e,f, respectively. Figure 2g is the histogram extracted from the peak position distribution from Figure 2f. The PL map for PeMOF thin film is reasonably uniform. The peak energy ranges from 2.55 to 2.65 eV with 0.1 eV variation across the testing area (50 µm by 50 µm). The even emission height map and narrow peak energy distribution of the Cs‐PeMOF thin film indicate that the nanocrystals are formed uniformly without significant aggregations. This is also validated by the TEM images shown in Figure S2 in the Supporting Information where separated nanocrystals can be observed across a large area.

**Figure 3:**
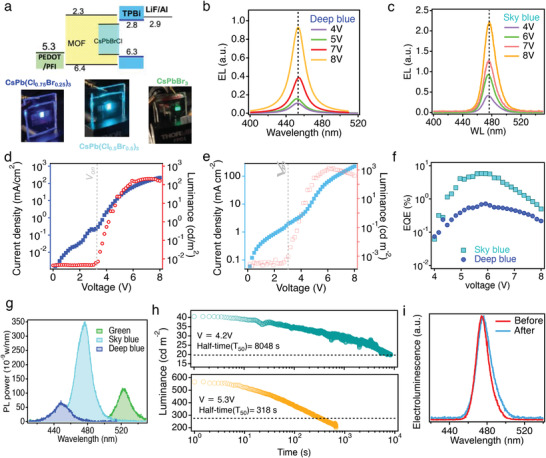
a) Energy alignment of the LED devices (top) and the photo of LEDs using PeMOF as emission layers (bottom). b,c) Electroluminescence spectra probed at various applied bias for deep blue and sky‐blue devices respectively. Current density (*J*) and luminance (*L*) as a function of applied voltage (*V*) for typical d) deep blue LED and e) sky blue LED. f) External quantum efficiency (EQE) for both PeMOF devices as a function of applied voltage. g) photoluminescence intensity comparison for sky‐blue, deep‐blue and green CsPeMOF thin films. Sky‐blue LED device stability study by h) recording the time evolution of the device's *L* when operated under constant bias, and i) comparing the EL spectra before and after the stability tests under high applied bias.

Following the thin film characterizations, we take the optimized PeMOF thin films for LEDs fabrications. The device structures utilized in this study is illustrated in **Figure** **3**a top panel, and the photographs of LEDs with deep blue, sky‐blue and green emissions assembled with Cs‐PeMOF thin films with various Br/Cl ratio are shown in the bottom panel. The energy alignment for Cs‐PeMOF thin films were obtained in Figure S11 in the Supporting Information. Figure 3b,c plot the electroluminescence (EL) spectra for deep blue and sky‐blue LEDs respectively, where the EL amplitude increases without noticeable peak shift when driven under various applied bias. Figure 3d,e shows the Current Density‐Luminance‐Voltage (*J*‐*L*‐*V)* characteristics for deep‐blue LED and sky‐blue LED made with PeMOF emission layers, and the LEDs’ figure‐of‐merits are summarized in **Table** **1**. Due to a higher injection current density, the peak luminance obtained from the sky‐blue device (1260 cd m^−2^) is significantly higher than that of the deep blue device (202 cd m^−2^). In addition, the sky‐blue device reaches a peak EQE value of 5.67% whereas the deep blue device only demonstrates an EQE of 0.72%. The higher EQE value indicates a more efficient radiative recombination of the injected electron/hole carriers in the sky‐blue device, which is ascribed to the better energy‐level alignment between the valence‐band maximum (VB_M_) of the Cs‐PeMOF and the highest occupied molecular orbital (HOMO) of the PEDOT:PFI layer. As evidenced by the ultraviolet photoelectron spectroscopy (UPS) spectra shown in Figure S11 in the Supporting Information, tuning the emission wavelength by increasing the Cl content will inevitably deepen the VB_M_ of the Cs‐PeMOF film, which may create a significant hole‐transporting energy barrier at the PEDOT:PFI/Cs‐PeMOF interface. Two other interface materials are investigated in Figure S12 in the Supporting Information, but the EL brightness is not further improved. To understand the EL performance, we have measured the photoluminescence quantum yield of the Cs‐PeMOF thin films in Figure 3g. The PLQY estimated from this experiment for the deep blue Cs‐PeMOF is 15% whereas the sky‐blue thin film is 54%, about 3 times higher than the deep blue thin film. The higher PLQY of the sky‐blue thin film mainly contributes to the higher EL efficiency of the LED. The reported PLQY for sky‐blue perovskite nanocrystal thin films ranges from 60% to 80% resulting in a high EL EQE of > 10%.^[^ ^18^, ^36^ ^]^ The relatively low PLQY for our Cs‐PeMOF thin film mainly contributes to the low EL EQE of ≈5.6%.

**Table 1:** Figure of merits for LEDs using two Cs‐PeMOF emission layers

PeMOF layer	Emission wavelength [nm]	FWHM [nm]	PLQY [%]	EQE [%]	Turn on voltage [*V*_ON_]	Brightness [cd m^−2^]
CsPb(BrCl)_1.5_	475	20.6	54	5.67	3.5	1260
CsPb(Br_1_Cl_2_)	452	23.9	15	0.72	3.8	202

After characterizing the LEDs’ performances, we take the brighter device made with sky‐blue PeMOF emitter for operational lifetime study. Here, we employ half lifetime (*T*_50_), the time when the device's luminance decays to 50% from its original value during constant operation, to evaluate the LEDs’ operation stability. Figure 3h shows the time evolution of the sky‐blue device under constant applied biases. As the results, at lower bias (4.2 V), the *T*_50_ of the device is 8048 s whereas the *T*_50_ is shortened (318 s) when higher bias (5.3 V) is applied. The shorter lifetime in blue LEDs than green LEDs are commonly observed and reported in PeLEDs and OLEDs which attribute to the constant charge injection induced defect formation.^[^ ^24^, ^37^ ^]^ We also collect the PL spectra in Figure 3i before and after the electrical stressing at high bias, the PL peak position of the Cs‐PeMOF device remains mostly unchanged, with a small tail towards lower energy appearing. This suggests that most of the nanocrystals don't undergo significant phase segregation.

To understand the stability mechanism in greater details, we have performed a power dependent photoluminescence characterization on the sky‐blue Cs‐PeMOF thin film to track the change in the PL peak position and intensity under various laser powers. We anticipate that when the laser power increases, the light induced phase segregation could occur at a faster rate. Therefore, by comparing the degradation rate of the PL intensity and peak position, the results will help us to understand the degradation mechanism of perovskites nanocrystals in MOF. Bulk CsPb(BrCl)_1.5_ thin film is also tested here for comparison.

**Figure 4:**
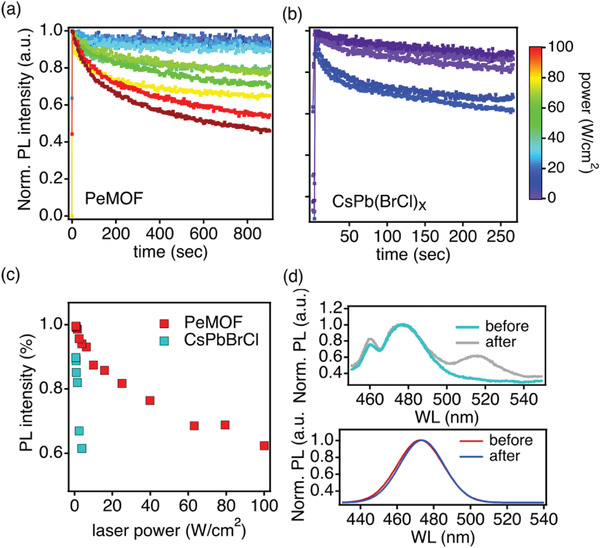
Stability investigation by photoluminescence. Time evolution of the PL intensity at 475 nm for a) Cs‐PeMOF emitter and b) bulk CsPb(BrCl)_1.5_ thin film when excited with different laser powers. c) Normalized PL intensity measured after 250 s of laser excitation for PeMOF and bulk CsPb(BrCl)_1.5_ thin film. d) The PL spectra before and after stability tests under constant 4 W cm^−2^ laser irradiation.

**Figure** **4**a,b plots the time evolution of the PL intensity change of both thin films. The laser powers used here are relatively high (0.5–100 W cm^–1^) to expedite the material degradation process. Under low laser power conditions (0.5–1 W cm^–1^), Cs‐PeMOF thin film can maintain over 98% of its original PL intensity whereas bulk CsPb(BrCl)_1.5_ thin film only maintains around 85%. As laser power increases, both thin films show PL intensity degradation, however, the Cs‐PeMOF thin film can stand for higher laser power irradiation before severe degradation occurs. This is reflected in Figure 4c where the percentages of the PL intensity degradation at the end of 250‐second testing period as a function of laser power are plotted. Clearly, the Cs‐PeMOF thin film can keep more than 90% of its original emission after 4 W cm^–2^ laser irradiation whereas the CsPb(BrCl)_1.5_ sample already losses 40% of the PL intensity. In addition, we compare the PL spectra for both samples before and after laser irradiation (4 W cm^–2^ in this case) are shown in Figure 4d. In Bulk CsPb(BrCl)_1.5_ thin film PL spectra, a new green emission feature (≈518 nm) appears after laser excitation which matches with the bulk CsPbBr_3_ emission peak, suggesting the phase segregation quickly happens after laser irradiation. In sharp contrast, the Cs‐PeMOF thin film maintains its original position with negligible peak shift. The mixed halide perovskites are well‐known to suffer from light or bias induced ion migration, phase segregation, where significant PL spectra shifts are observed. Based on our PL stability tests under constant laser irradiation, the Cs‐PeMOF thin film can stand for higher laser power before the PL degradation occurs, and the PL spectrum for this sample has negligible changes after the light stress at high laser power density.

In addition to the light stability, the resilience of a perovskite material under the operational electric filed is also crucial for LEDs applications. In general, a distinctive halide component gradient can be readily observed after an intense voltage or current stressing due to the large mobility of the halide ion. The mobile halide ions in the perovskite layers have been reported to induce severe device degradations via different mechanisms, e.g., electrode corrosions^[^ ^38^ ^]^ or space charge accumulations.^[^ ^39^ ^]^ To further verify the robustness of the Cs‐PeMOF nanocrystals for LED applications, ion migration induced PL we perform time‐of‐flight secondary ion mass spectrometry (TOF‐SIMS) depth profiling measurements on the aged Cs‐PeMOF film after PL irradiation and the results are summarized in Figure S13 in the Supporting Information. TOF‐SIMS is a recognized feasible technique to probe the ion migration effect in hybrid perovskite thin film and devices under various test conditions.^[^ ^38^, ^40^ ^]^ From the TOF‐SIMS depth profiling data, we can identify most positive specious in Cs‐PeMOF thin film. The Pb, Cs and Si can be observed in (+) ion mode, and Cs2Br, Cs2Cl, and CsC6H3 can be obtained in (+) ion mode as well. In the aged Cs‐PeMOF thin film samples, all the observed specious are uniformly distribute across the whole layer thickness, suggesting the perovskite nanocrystal and MOF matrix are preserved after PL irradiation. It is observed that both the cation and anion components are uniformly distributed throughout the film, validating the suppressed diffusion dynamic of the perovskite component within the MOF matrix. Accordingly, the restricted component mobility enabled by the PeMOF nanocrystals will remarkably benefit the operational stability of the LEDs device.

## 3 Discussion

It is known that CsPb(Br*_x_* Cl_3−_ *_x_*) mixed halide nanocrystals undergo phase segregation that shift the emission spectra under stress conditions such as constant laser irradiation and current injection.^[^ ^25^ ^]^ This is attributed to the field induced ion migration that causes phase segregation.^[^ ^26^ ^]^ And the ion migration is believed to initiate near the defect site, and halide vacancies are identified to be the main thermodynamically favorable defects formed in a perovskite structure. In particular, the Cl and Br ions diffuse at a relatively fast rate than the I ions, which makes it more challenging to stabilize the blue perovskite LEDs.^[^ ^28^ ^]^

In our prior study,^[^ ^5^ ^]^ we found that the perovskite nanocrystals in MOF matrix (PeMOF) exhibit lower defect density, stronger emission yield and large degree of charge localization. These results indicate that the nanocrystals assembled in the MOF matrix are free from halide vacancies. Similarly, in the Cs‐PeMOF case studied in this work, we found the CsPbX_3_ nanocrystals are more robust under the constant laser irradiation, and the phase segregation does not occur until the laser power is over certain threshold. This can be attributed to the cleaner surfaces of the CsPbX_3_ nanocrystals, the surface defects are passivated by the MOF linker resulting in a lower defect density.

Another point worth discussing is the observed discrepancy between the nanophase segregation observed in the TEM image and the lack of the green emission in the PL spectrum. We consider two possibilities here. First, the phase segregation is in nanoscale, we hypothesize that charge or energy transfer could occur across the nanophases because the distance between the two crystals are less than 10 nm, where the emission color is from an intermediate charge transfer state. In order to probe this possibility, we performed time resolved PL for the alloy CsPb(Cl/Br)_1.5_ nanocrystal (blue) and CsPbBr_3_ nanocrystals (green). Both thin films are assembled in the MOF matrix fabricated via the same fabrication method. The results are shown in Figure S8 in the Supporting Information. In both samples, the PL decay rapidly that is commonly observed in perovskite nanocrystals where carriers are strongly bonded. Interestingly, the sky‐blue Cs‐PeMOF decays faster than that of the green Cs‐PeMOF. Also, we found that in 1 ns after the laser pulse, the PL signal from the sky‐blue Cs‐PeMOF raises slowly whereas that from the green Cs‐PeMOF immediately reaches a maximum within 0.5 ns. The slower raise time of the sky‐blue Cs‐PeMOF could originate from charge transfer.

However, one cannot eliminate another possibility that's related to the measurement tool induced sample degradation. It is possible that the TEM damage is more significant than the laser irradiation therefore we observe a phase segregation in the TEM image, but no green emission from the PL spectrum. This point worth further investigation.

## 4 Conclusion

In conclusion, we demonstrate all inorganic halide perovskite nanocrystals in MOF thin films, which can protect the nanocrystals from merging and degradation. The perovskite nanocrystals are well separated by the MOF matrix. By tuning the ratio of chloride and bromide in the precursor, the thin films exhibit deep blue or sky‐blue emissions. The thin film can be used as emission layers for light emitting diodes where bright electroluminescence is demonstrated. Notably, the EL intensity and spectra stability are improved using the MOF protected perovskite nanocrystal layers. By detailed PL stability and TOF‐SIMS characterizations, the perovskite‐MOF thin film exhibits much higher threshold for light induced phase segregation and material degradation.

## 5 Experimental Section

### PeMOF Thin Film and Device Characterization

The Pb‐MOF material was synthesized follow by the previous reported method.^[^ ^33^ ^]^ The Pb(NO_3_)_2_ (10 × 10^−3^ m, 50 mL) was dissolved in de‐ionic water and 1,3,5‐H_3_BTC (10 × 10^−3^ m, 50 mL) was dissolved in ethanol in two separated beakers, then transfer the Pb(NO_3_)_2_ beaker to ultrasonic bath with slowly added the 1,3,5‐H_3_BTC solution with 30 min of sonication. The white precipitations (Pb‐MOF) were filtered and washed with ethanol before vacuumed dried the solvent residue. The Pb‐MOF thin film was spun cast the Pb‐MOF solution (in DMF with trace amount of HBr or HCl) with 5K r.p.m for 30 s and heated for 20 min at 120 °C. Various CsBr/CsCl molar ration was spun cast (5K r.p.m., 30 s) on the Pb‐MOF thin film. The fabricated thin film was post treated at 100 °C for 10 min for remove solvents. Absorption spectra of the thin films were measured using a UV‐visible spectrometer (V730, JASCO). PL and PL mapping measurements of the films were obtained using a confocal laser microscope. The device structure was ITO/PEDOT:PSS/PFI/PeMOF/TPBi/LiF/Al where PEDOT:PSS/PFI were spun cat with 5K rpm for 30 s with post annealed at 100 °C for 20 min and TPBI(30 nm)/LiF(1 nm)/Al(100 nm) were deposited using thermal evaporation.

### GIWAXS Characterization

The synchrotron GIWAXS samples were placed in the rotary sample stage under medium vacuum (10^−3^ torr) and exposed to an X‐ray beam (*λ* = 1.13538 Å or 10.92 keV) at an incident angle of 0.14° for 5 s, and the scattered photons were collected by a Pilatus 1 M pixel array detector at 217 mm from the sample. The crystalline domain size was obtained from fitting the peaks’ FWHM as a function of *q*, according to the Scherrer equation after experimental resolution correcting.^[^ ^41^ ^]^

### TEM Characterization

High resolution transmission electron microscopy (HRTEM) was performed on a monochromated and aberration corrected FEI Titan operating at 300 keV. Images were captured using a Gatan K2 direct detection camera.

### AFM Analysis

The surface topography of the Cs‐PeMOF thin film was identified by a Veeco Innova SPM system under the tapping mode.

### XPS Analysis

All the XPS spectra were acquired by a PHI 5000 VersaProbe (ULVAC‐PHI, Japan) system. A focused monochromatic Al K*α* radiation was utilized as the X‐ray source to generate the characteristic photoelectrons of the investigated elements. During the XPS measurements, low‐energy electron and Ar^+^ flooding were applied to compensate the surface charge on the sample.

## Conflict of Interest

The authors declare no conflict of interest.

## Supporting information

Supporting Information

## Data Availability

The data that support the findings of this study are available from the corresponding author upon reasonable request.
