# Supplementary material for: Cesium Lead Halide Perovskite Nanocrystals Assembled in Metal‐Organic Frameworks for Stable Blue Light Emitting Diodes
Source: Adv Sci (Weinh). 2022 Mar 15;9(14):2105850. doi: 10.1002/advs.202105850 (PMC9108663; doi:10.1002/advs.202105850)
Supplement: Supplementary file 1 — Supporting Information [file ADVS-9-2105850-s001.pdf]

Supporting Information

Cesium lead halide perovskite nanocrystals assembled in metal-organic frameworks for stable blue light emitting diodes

Hsinhan Tsai, Hsin-Hsiang Huang, John Watt, Cheng-Hung Hou, Joseph Strzalka, Jing-Jong Shyue, Leeyih Wang and Wanyi Nie**

Dr. Hsinhan Tsai, Dr. John Watt, Dr. Wanyi Nie
Center for Integrated Nanotechnologies, Los Alamos National Laboratory, Los Alamos, NM, 87545, United States
E-mail: wanyi@lanl.gov

Dr. Hsinhan Tsai
Department of Chemistry, University of California, Berkeley, Berkeley, California 94720, United States
E-mail: hsinhantsai@berkeley.edu

Dr. Hsin-Hsiang Huang, Prof. Leeyih Wang
Center for Condensed Matter Sciences, National Taiwan University, Taipei 10617, Taiwan

Dr. Hsin-Hsiang Huang
Department of Material Science and Engineering, National Taiwan University, Taipei 10617, Taiwan

Prof. Leeyih Wang
Center of Atomic Initiative for New Materials, National Taiwan University, Taipei, 10617, Taiwan

Dr. Joseph Strzalka
X-Ray Science Division, Argonne National Laboratory, Argonne, IL 60439, United States

Dr. Cheng-Hung Hou, Dr. Jing-Jong Shyue
Research Center for Applied Science, Academia Sinica, Taipei 11529, Taiwan

Keywords: inorganic perovskite nanocrystals, blue LEDs, Metal-Organic Frameworks

Table S1 Summary of the reported blue LED demonstrations.

**Material**	**EL peak (nm)**	**FWHM (nm)**	**brightness (Cd/m2)**	**EQE (max)**	**Lifetime (min)**	**Driving condition**	**Ref**
PEABr incorporated $CsPbCl_xBr_{3-x}$	480	20	3780	5.7%	T50=10min	V=4.4Volt<br>J=0.1 mA/cm^2	[1]
$CsPbCl_xBr_{3-x}$ NC	475		350	0.07%			[2]
Quasi-2D $PEA_2A_{1.5}Pb_{2.5}Br_{8.5}$	490	28	2480	1.05%	T50=10min	L0=10 cd/m^2	[3]
CsPb(Mn)Cl/Br NC<br>$CsMnPb(Br_{1-x}Cl_x)_3$	470<br>466	17.9	389<br>245	1.15%<br>2.12%			[4]
3D $Cs_{10}(MA_{0.17}FA_{0.83})_{100-x}PbBr_{1.5}Cl_{1.5}$	475	28	3564	1.7%	T25=150min	J=3 mA/cm^2<br>L0=345 cd/m^2	[5]
Quasi-2D $(EA)_2(MA)_{n-1}Pb_nBr_{3n+1}$	473<br>485	>20nm	200	2.6%	T50=10~20 min	L0=100 cd/m^2<br>J=10~25 mA/cm^2	[6]
Quasi-2D $CsPbBr_3$ RP perovskites	486		3340	6.2%			[7]
$CsPbCl_xBr_{3-x}$ QD	490	19	35	1.9%			[8]
$Cs_xFA_{1-x}PbBr_3$ NCs with $CuCl_2$	490	19	1946	5.02%			[9]
Quasi-2D $(PEA)CsPbX_3$+Na	488		1000	11.7%	T50=15min	L0=100cd/m^2	*[10]*
Ni^{2+} ion-doped $CsPbCl_{0.99}Br_{2.01}$ PQDs	470		612	2.4%			*[11]*
Quasi-2D PEA/iBA	485		1130	7.84%	T50=5min	L0=100 cd/m^2	[12]
$PEA_2(Cs_{1-x}EA_xPbBr_3)_2PbBr_4$	488	25	2191	12.1%	T50=10min	L0=100cd/m^2	[13]
$CsPbBr_3$ QD	479	20	10000	12.3%	T50=20 min	L0=30 cd/m^2	[14]

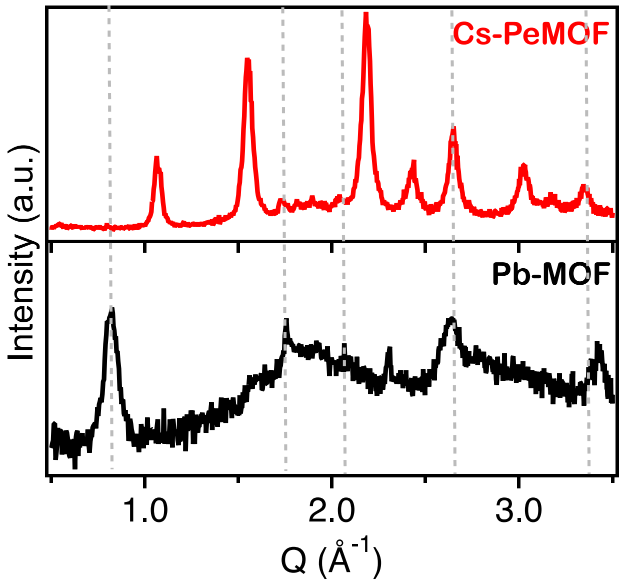

Figure S1. GIWAXS line-cut comparison for Pb-MOF (Figure 1c) and Cs-PeMOF (Figure 1d). The dashed lines indicate the MOF structures that are preserved in the PeMOF thin film.

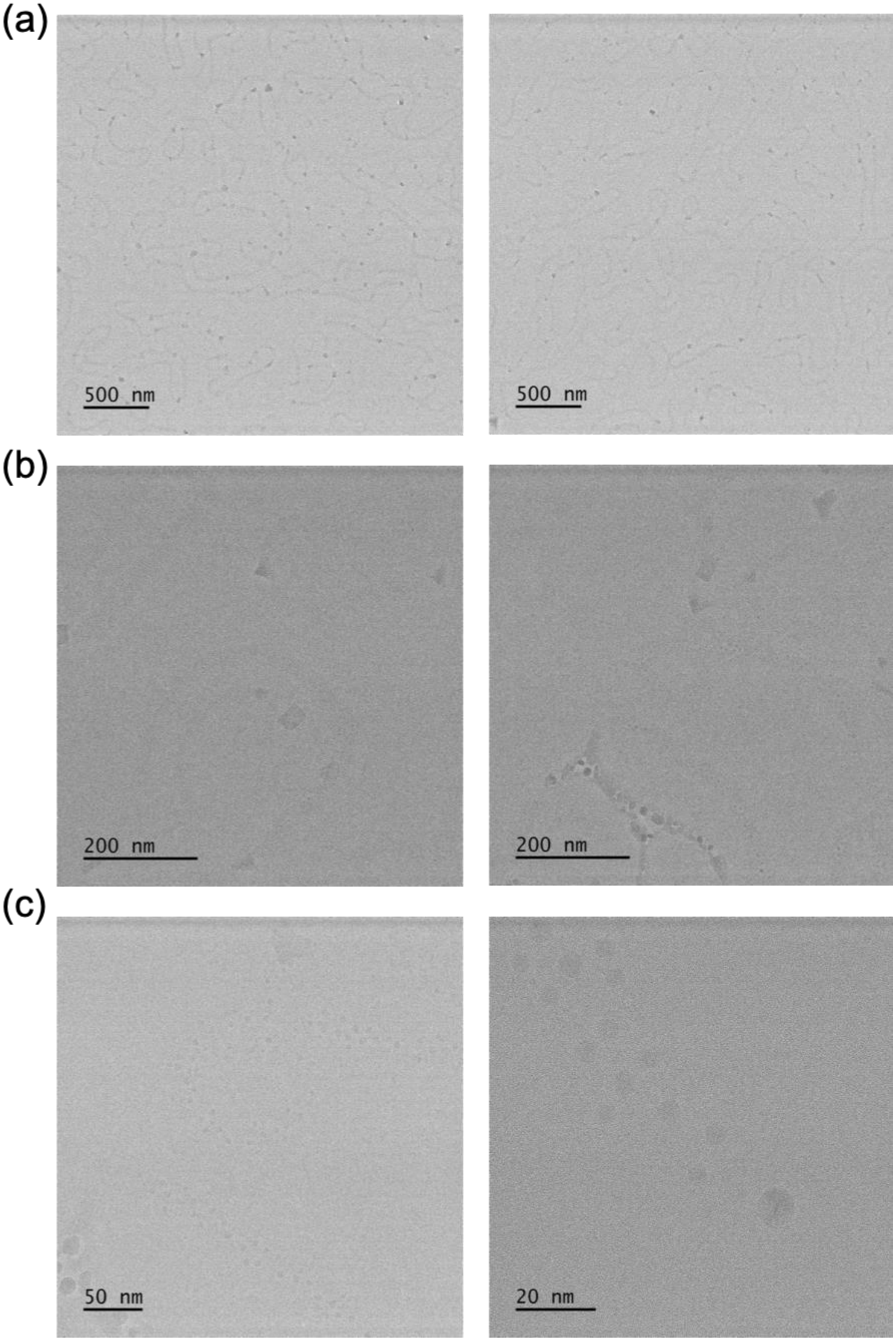

Figure S2. Transmission electron microscopy images for several samples with different magnifications. Clusters of nanocrystals can be observed in Fig. S1(b). Within the cluster, the crystals are still well separated. The sizes of the crystals are found to be in the range of 10-20 nm.

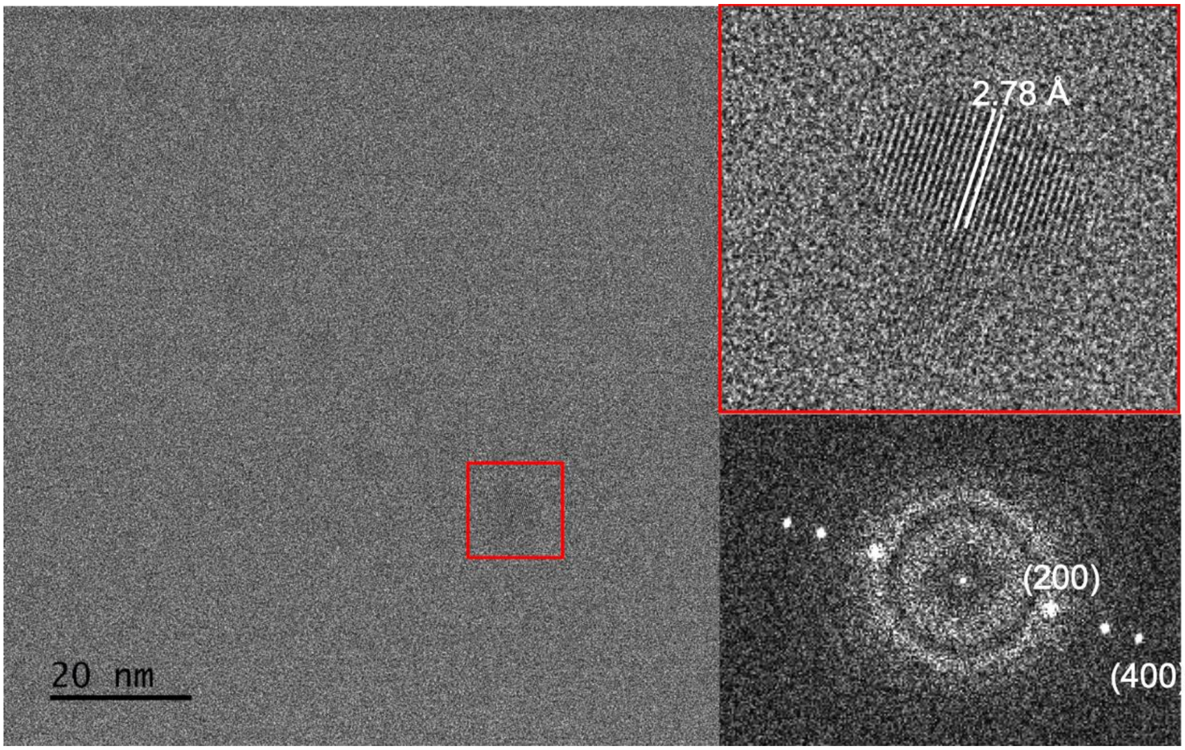

Figure S3. Analysis on one typical $CsPbX_3$ nanocrystal in the MOF matrix. The lattice spacing in this crystal is found to be 2.78 Å, which matches with $CsPbCl_3$ Tetragonal P4mm (99). The (200) and (400) peaks can be also found in the electron diffraction pattern.

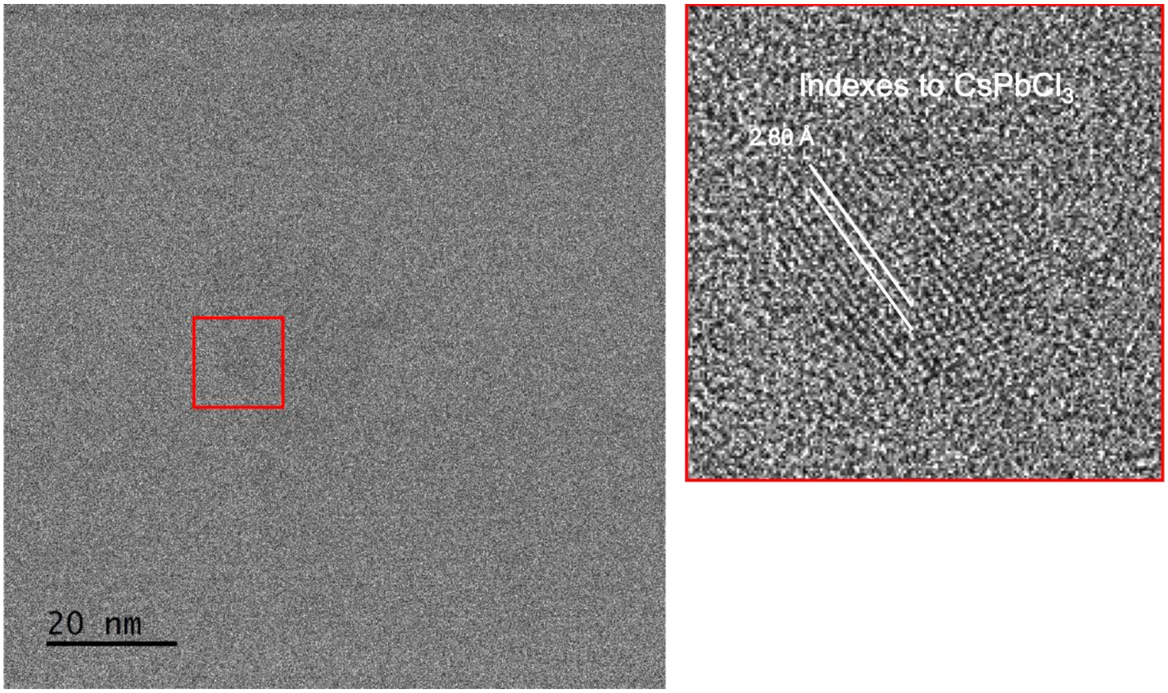

Figure S4. Analysis on one typical $CsPbX_3$ nanocrystal in the MOF matrix. The lattice spacing is found to be 2.86 Å, that best matches the (200) of $CsPbCl_3$ Tetragonal P4mm(99)

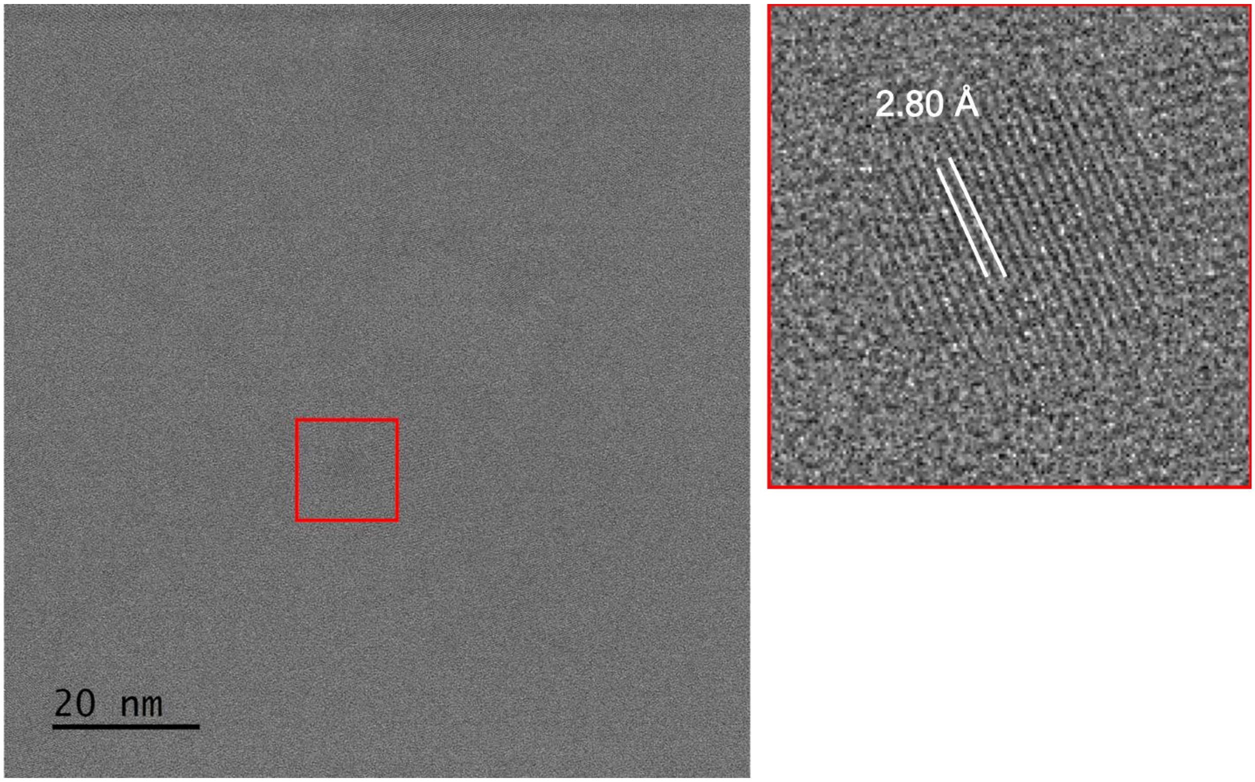

Figure S5. Analysis on one typical $CsPbX_3$ nanocrystal in the MOF matrix. The lattice spacing is found to be 2.80 Å, that best matches the (200) of $CsPbCl_3$ Tetragonal P4mm(99)

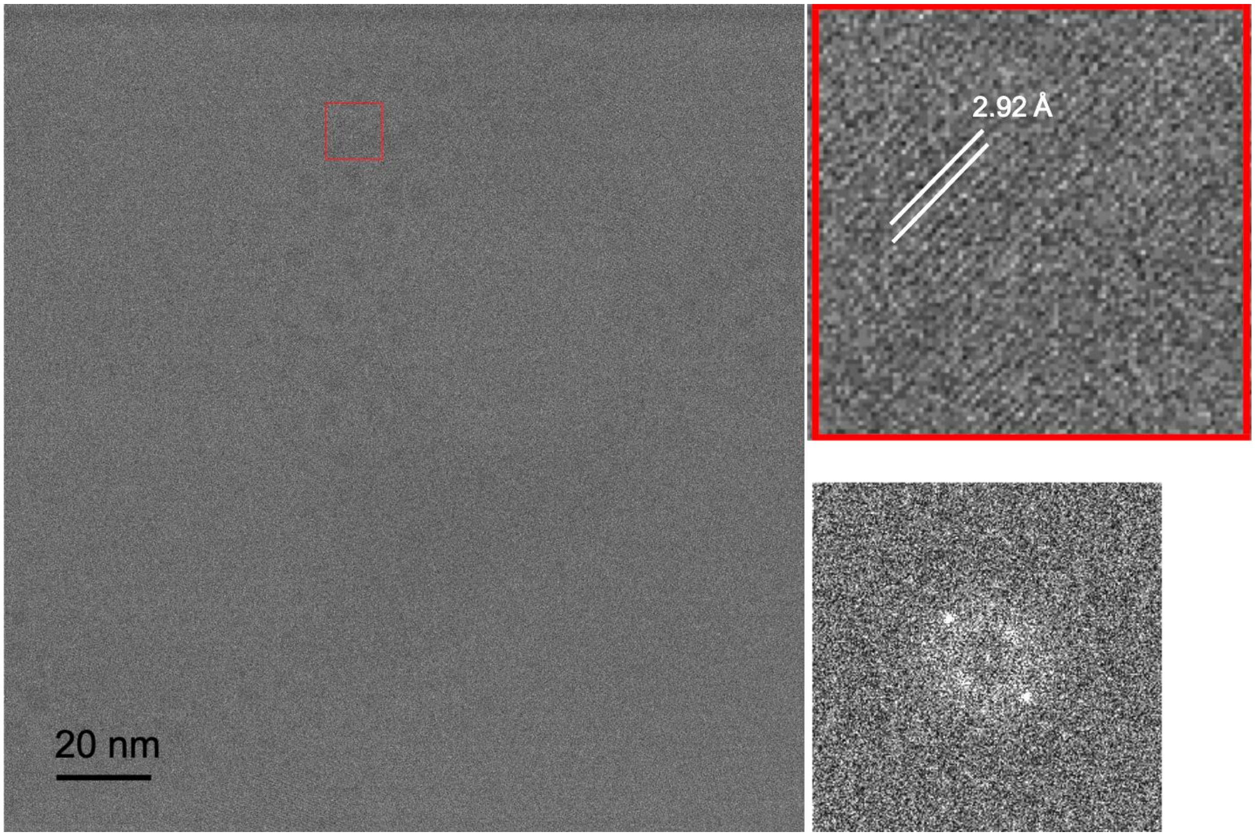

Figure S6. Analysis on one typical $CsPbX_3$ nanocrystal in the MOF matrix. The lattice spacing is found to be 2.92 Å, that best matches the (202) of $CsPbBr_3$ Orthorhombic Pnma (62).

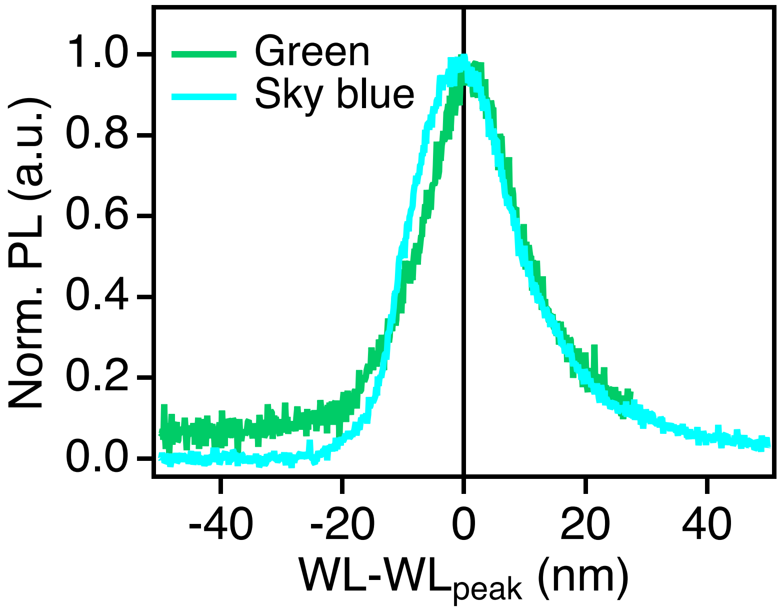

Figure S7. PL spectra for CsPbBr3 PeMOF (green) and $CsPb(BrCl)_{1.5}$ PeMOF (sky blue) with normalized at peak position and intensity.

It's well known that PL line width is high affected by material quality and compositions. Here we revisit the PL spectra for $CsPbBr_3$ PeMOF (green) and $CsPb(BrCl)_{1.5}$ PeMOF (sky blue) with normalized at peak position (X-axis) and intensity (Y-axis) as shown in Fig. S7. From PL spectra, the $CsPbBr_3$ PeMOF (green) exhibit much symmetry peak shape with full width at half maximum (FWHM) of 20.24 nm whereas $CsPb(BrCl)_{1.5}$ PeMOF has asymmetry peak shape with slightly increase in FHWM (20.64 nm). This suggested that the mix-halides $CsPb(BrCl)_{1.5}$ PeMOF is not as stable phase as $CsPbBr_3$ PeMOF, however, still have comparable material crystallinity.

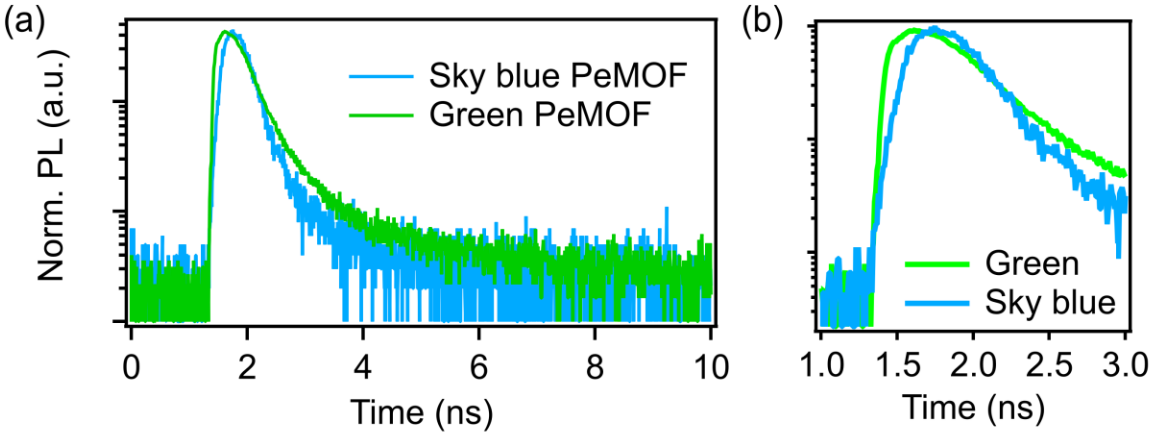

Figure S8 (a) Normalized time evolution of the PL decay for sky blue and green Cs-PeMOF. (b) Zoom-in view of the early time regime to compare the rise time.

We first study the PL lifetime of the thin films by comparing the sky-blue Cs-PeMOF with green Cs-PeMOF thin films. Figure R2 plots the TRPL results, both Cs-PeMOF samples show rapid decays within 10 ns, the short lifetime is related to the strong carrier binding energy in the nano-crystals. Interestingly, the sky-blue Cs-PeMOF thin film has a slower rise time and a faster decay time than that of the green Cs-PeMOF sample. The shorter lifetime can be either attributed to a faster radiative recombination promoted by carrier localization, or trap assisted recombination.

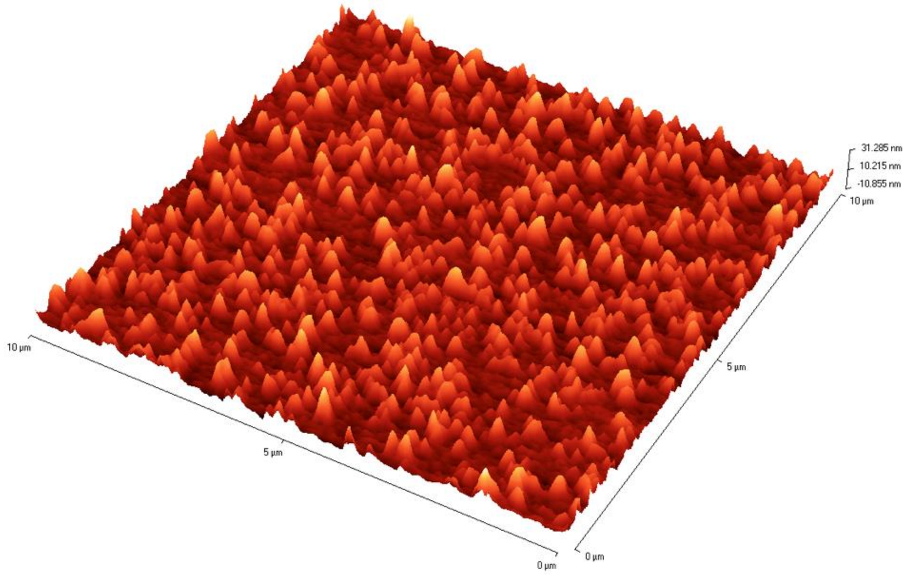

Figure S9. AFM image for a Pb-MOF thin film. The roughness is estimated to be 4.96 nm.

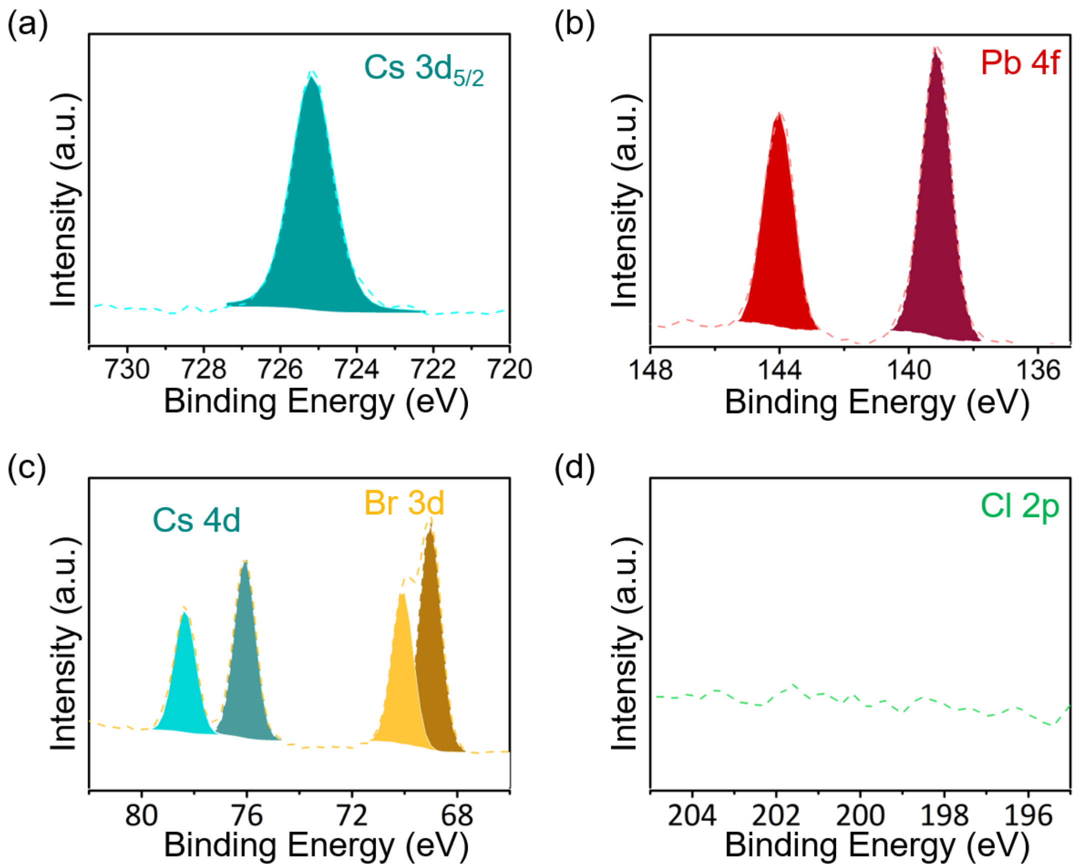

Figure S10. Characteristic XPS spectra of (a) Cs, (b) Pb, (c) Br and (d) Cl elements obtained from pure bromide $CsPbBr_3$-PeMOFs film.

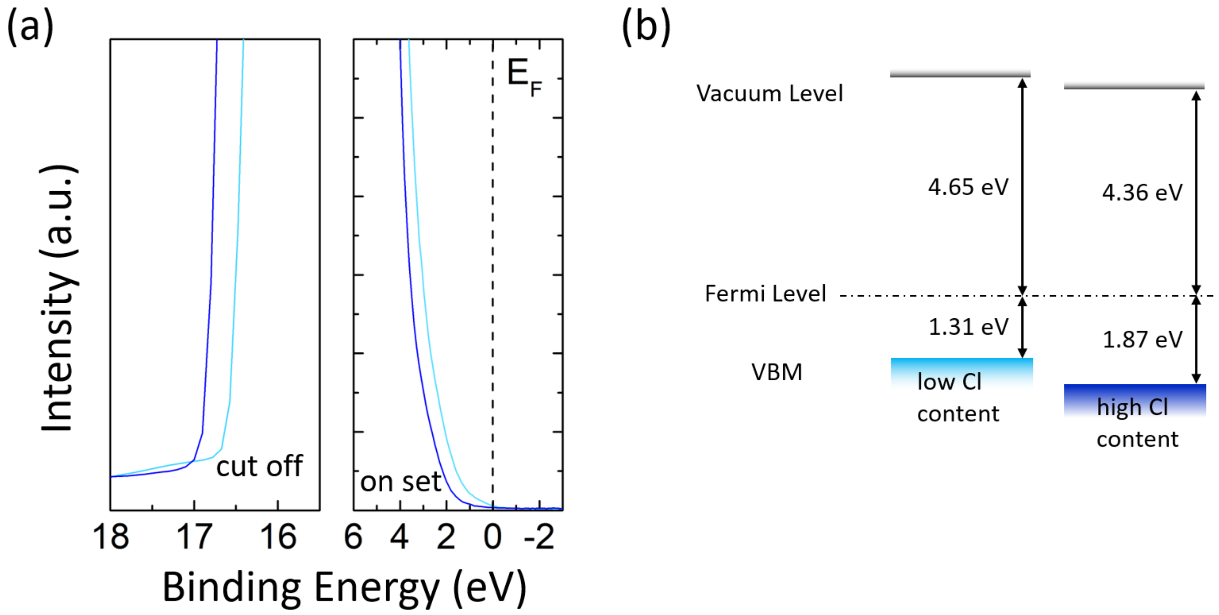

Figure S11. (a) UPS spectra and (b) corresponding energy-level diagrams of the Cs-PeMOF films with different Cl contents.

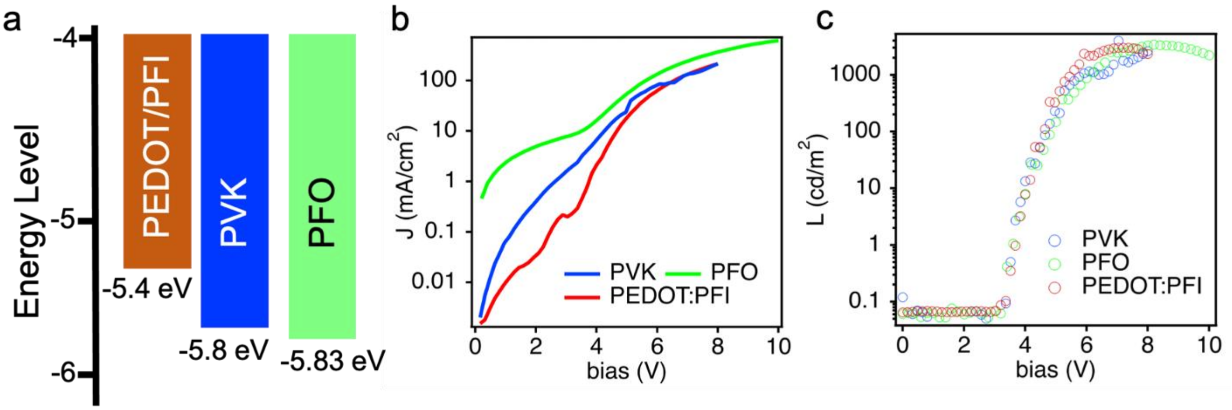

Fig. S12 a, energy alignment for three different hole injection layer (HIL) materials. b, current density (J) and (c) luminance (L) as a function of bias for the three devices using these HILs.

We have performed additional experiments comparing two additional hole injection layer (HIL) materials: Poly(9-vinylcarbazole) (PVK) and Poly(9,9-di-n-octylfluorenyl-2,7-diyl) (PFO) which are commonly used for perovskite LEDs. The energy diagram for these hole injection layer materials is shown in Fig. S1a, both PVK and PFO have deeper VBM than the currently used PEDOT/PFI hole injection layer. The current voltage and luminance curves are summarized in Fig. S11b-c.

Interestingly, when replacing the PEDOT/PFI with PVK increase the low bias injection current and the saturation current at higher bias merges with the PEDOT/PFI device. When using PFO as the HIL, the low bias current further increase and the high injection current is also higher than the other two devices. The low injection current increase when using PVK could be attributed to the deeper VBM that aligns better with the perovskite material. However, the large injection current increase when using PFO at lower bias is attributed to recombination due to interface electronic traps. Therefore, even though the injection currents were higher when using PFO and PVK as the injection layer, the emission brightness shown in Fig. S11c wasn’t improved. This suggests that the excess injected carriers undergo non-radiative recombination in the device, either due to trap assisted recombination or direct recombination at the other interface

(e.g., electron injection interface) because of the imbalanced charge injection. From these comparisons, we can conclude that the interface energy alignment plays a minor role in the device performance.

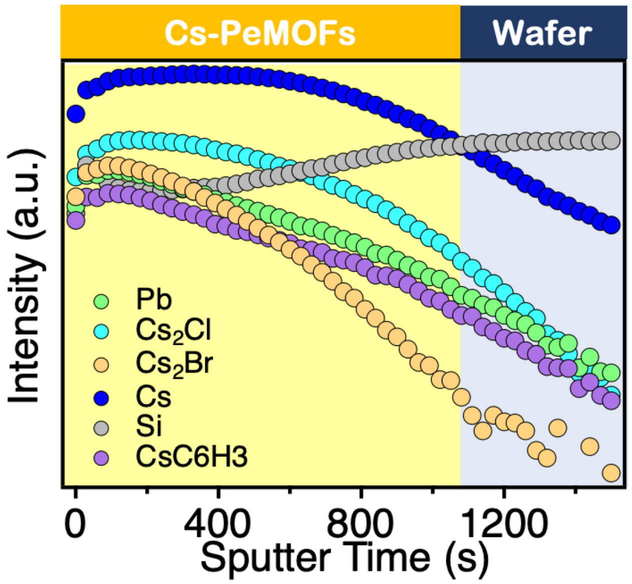

Figure S13. Time flight secondary ion mass spectroscopy (TOF-SIMS) result for the PeMOF thin film after laser irradiation.

ToF-SIMS Depth Profiling. The Cs-PeMOF samples for ToF-SIMS measurement were fabricated on pre-clean wafer followed the same method aboved. The ToF-SIMS depth profile is acquired using a PHI TRIFT V nanoTOF (ULVAC-PHI, Japan) system by the dual beam slice-and-view scheme. A pulsed 20 kV-C_{60}^+ ion (approximately 8200 Hz, 15 ns pulse length) and a 500 V-Ar^+ ion is used as the primary ion and the sputter ion, respectively. During the spectrum acquisition and the surface sputtering step, low-energy electron and Ar^+ flooding are applied to compensate the surface charge. To compensate the possible fluctuations of the primary ion beam current, all the signal intensities shown in the ToF-SIMS depth profile are normalized by the total ion intensity.

References

[1]Z. Li, Z. Chen, Y. Yang, Q. Xue, H.-L. Yip, Y. Cao, Nature Communications 2019, 10, 1027.

[2] E.-P. Yao, Z. Yang, L. Meng, P. Sun, S. Dong, Y. Yang, Y. Yang, Advanced Materials 2017, 29, 1606859.

[3] J. Xing, Y. Zhao, M. Askerka, L. N. Quan, X. Gong, W. Zhao, J. Zhao, H. Tan, G. Long, L. Gao, Z. Yang, O. Voznyy, J. Tang, Z.-H. Lu, Q. Xiong, E. H. Sargent, Nature Communications 2018, 9, 3541.

[4] S. Hou, M. K. Gangishetty, Q. Quan, D. N. Congreve, Joule 2018, 2, 2421.

[5] H. P. Kim, J. Kim, B. S. Kim, H.-M. Kim, J. Kim, A. R. b. M. Yusoff, J. Jang, M. K. Nazeeruddin, Advanced Optical Materials 2017, 5, 1600920.

[6] Q. Wang, J. Ren, X.-F. Peng, X.-X. Ji, X.-H. Yang, ACS Applied Materials & Interfaces 2017, 9, 29901.

[7] P. Vashishtha, M. Ng, S. B. Shivarudraiah, J. E. Halpert, Chemistry of Materials 2019, 31, 83.

[8] J. Pan, L. N. Quan, Y. Zhao, W. Peng, B. Murali, S. P. Sarmah, M. Yuan, L. Sinatra, N. M. Alyami, J. Liu, E. Yassitepe, Z. Yang, O. Voznyy, R. Comin, M. N. Hedhili, O. F. Mohammed, Z. H. Lu, D. H. Kim, E. H. Sargent, O. M. Bakr, Advanced Materials 2016, 28, 8718.

[9] F. Chen, L. Xu, Y. Li, T. Fang, T. Wang, M. Salerno, M. Prato, J. Song, Journal of Materials Chemistry C 2020, 8, 13445.

[10] P. Pang, G. Jin, C. Liang, B. Wang, W. Xiang, D. Zhang, J. Xu, W. Hong, Z. Xiao, L. Wang, G. Xing, J. Chen, D. Ma, ACS Nano 2020, 14, 11420.

[11] G. Pan, X. Bai, W. Xu, X. Chen, Y. Zhai, J. Zhu, H. Shao, N. Ding, L. Xu, B. Dong, Y. Mao, H. Song, ACS Applied Materials & Interfaces 2020, 12, 14195.

[12] F. Wang, Z. Wang, W. Sun, Z. Wang, Y. Bai, T. Hayat, A. Alsaedi, Z. a. Tan, Small 2020, 16, 2002940.

[13] Z. Chu, Y. Zhao, F. Ma, C.-X. Zhang, H. Deng, F. Gao, Q. Ye, J. Meng, Z. Yin, X. Zhang, J. You, Nature Communications 2020, 11, 4165.

[14] Y. Dong, Y.-K. Wang, F. Yuan, A. Johnston, Y. Liu, D. Ma, M.-J. Choi, B. Chen, M. Chekini, S.-W. Baek, L. K. Sagar, J. Fan, Y. Hou, M. Wu, S. Lee, B. Sun, S. Hoogland, R. Quintero-Bermudez, H. Ebe, P. Todorovic, F. Dinic, P. Li, H. T. Kung, M. I. Saidaminov, E. Kumacheva, E. Spiecker, L.-S. Liao, O. Voznyy, Z.-H. Lu, E. H. Sargent, Nature Nanotechnology 2020, 15, 668.
